# Antibiotic treatment of *Chlamydia*-induced cystitis in the koala is linked to expression of key inflammatory genes in reactive oxygen pathways

**DOI:** 10.1371/journal.pone.0221109

**Published:** 2019-08-15

**Authors:** Samuel Phillips, Bonnie L. Quigley, Ammar Aziz, Wendy Bergen, Rosemary Booth, Michael Pyne, Peter Timms

**Affiliations:** 1 Genecology Research Centre, Faculty of Science, Health, Education and Engineering, University of the Sunshine Coast, Queensland, Australia; 2 Menzies School of Health Research, Charles Darwin University, Darwin, Northern Territory, Australia; 3 Australia Zoo Wildlife Hospital, Steve Irwin Way, Queensland, Australia; 4 Currumbin Wildlife Hospital, Currumbin, Queensland, Australia; University of California, San Francisco, Universit of California, Berkeley and the Childrens Hospital Oakland Research Institute, UNITED STATES

## Abstract

Chlamydial-induced cystitis in the koala (*Phascolarctos cinereus*) is currently treated by antibiotics. However, while reducing the chlamydial load, this treatment can also lead to gastrointestinal complications and death. Development of alternative treatments, such as a therapeutic chlamydial vaccine, are hindered by the lack of detailed understanding of the innate immune response to chlamydial clearance and disease regression during antibiotic treatment. Through clinical, microbiological and transcriptomic approaches, disease regression, bacterial clearance and innate immune responses were mapped in koalas with signs of chlamydial-induced cystitis while receiving anti-chlamydial antibiotics. Significant reduction in the signs of cystitis were observed during and post antibiotic treatment. This was observed as a thinning of the bladder wall and complete reversal of urinary incontinence. Transcriptomic analysis before treatment, at the end of treatment and prior to release identified significant down-regulation of specific genes involved in 21 biological pathways. Of these, the chemokine receptor signalling and NOD-like receptor signalling pathways where identified as important markers of inflammation. Specific genes within these pathways (*NCF1* and *NOX2*) were significantly down-regulated, suggesting a decrease in reactive oxygen species production. Through the monitoring of specific clinical and transcriptomic markers, these findings allow detailed profiling of the clinical response to therapeutic vaccination in koalas with current signs of disease. This also adds to our understanding of innate immune responses to chlamydial infections and indicates that chlamydial-induced cystitis in the koala is linked to the regulation of reactive oxygen pathways.

## Introduction

Cystitis is the inflammation of the uroepithelium within the bladder wall due to either biological or mechanical injury. In the case of bacterial infections, the basement membrane of the uroepithelium becomes damaged due to bacterial toxins and bacterial intracellular infiltration through the cell wall. Other factors affecting the uroepithelium during cystitis include neutrophil-associated damage (as a result of the innate immune response) and dysregulation of pro-inflammatory cytokines (including interleukin (*IL*) *6*, *IL1β* and *IL8*) [1–3]. In chronic infections, the involvement of monocytes and macrophages is stimulated via tumour necrosis factor signalling pathways and tissue resident monocytes to aid in the phagocytosis of pathogenic bacteria and cellular debris [4]. *Chlamydia* infections of the uroepithelium are common and have been studied in both mice and guinea pigs. These studies have observed that the regulation and activation of the cytokine *IL1β* plays an important role in infection outcomes [5–7]. However, progression of these urethral infections to cystitis is rarely documented and clearance of bacterial shedding often occurs without intervention.

Unlike other *Chlamydia* species, *Chlamydia pecorum* infections of the uroepithelium in the Australian marsupial, *Phascolarctos cinereus* (koala), commonly ascends the urinary tract leading to the development of chronic cystitis [8, 9]. Chlamydial infections in free-ranging koalas in southeast Queensland are endemic, with disease rates as high as 30% of surveyed animals [8, 10]. Infections and disease of the urogenital tract account for approximately 87% of all infections in some koala populations and persist for extended periods of time [9–11]. In the absence of antibiotic intervention, these persistent infections can result in the development of cystitis (observed as urinary incontinence and thickened bladder wall), resulting in kidney involvement and the development of nephritis [8, 9, 12]. Another complication of urogenital tract infection is reproductive tract disease, with reproductive infections resulting in ovarian bursal cysts and infertility [11, 13]. In addition to urogenital tract infections, *C*. *pecorum* can also infect the conjunctiva and gastrointestinal tract of koalas, with ocular infections resulting in scarring and eventual blindness [14, 15].

*Chlamydia* infections in the koala are effectively treated with antibiotics for extended periods of time (up to 45 days) [8, 11]. Unfortunately, antibiotic treatment commonly leads to gastrointestinal dysbiosis, which results in the wasting, and eventual death, of the koala [16, 17]. This poor outcome has driven the development of a therapeutic vaccine to combat *C*. *pecorum* infections in the koala. Vaccine trials have shown positive results in the reversal of ocular pathology and the stimulation of an adaptive immune response [18, 19]. The mechanisms of disease regression are poorly understood in koala chlamydial infections of the uroepithelium. A detailed understanding of the immunological responses present during cystitis regression will enable the monitoring of immune pathways active during vaccination and aid researchers in defining therapeutic advantages.

This koala chlamydial study provides evidence for the down-regulation of *IL1β* activation through the regulation of specific genes in reactive oxygen pathways during antibiotic treatment for chlamydial-induced cystitis. These findings add to the knowledge of the innate immune responses to chlamydial infections and indicate a possible mechanism involved in chlamydial disease regression in other hosts and tissues.

## Methods

### Animal trial

Five Koalas, two male and three female (between three and five years of age), that presented to Currumbin Wildlife Hospital (CWH) or Australia zoo wildlife hospital (AZWH) with signs of cystitis were included in the trial. The animal’s disease grade was determined using ultrasound and visual inspection while the koalas overall health status was determined using visual and physical inspection. All koalas from CWH were transported to AZWH for treatment and re-examined on arrival (as above) to confirm the diagnosis of cystitis.

Duplicate urogenital, rectal and ocular swabs were collected before antibiotics were administered. Antibiotics used were: Doxycycline, 5 mg/kg diluted 50:50 in sterile saline, given subcutaneously, once per week for four weeks or chloramphenicol, 60 mg/kg given subcutaneously once per day for 28 days. Every two weeks, koalas were examined and had duplicate swabs collected from the urogenital, rectal and ocular sites. This regimen was continued for eight weeks, with the exception of two koalas that were euthanised at two and six weeks. Further to antibiotic treatment, the occasional use of the steroidal injections (prednisolone) was used to aid in healing and reduce inflammation. Of the five koalas in the trial, two received the steroid, prednisolone. One female for three days (until euthanised) and one male for 17 days. Furthermore, one female koala received sub-conjunctival treatment with methylprednisolone on three separate occasions due to severe conjunctival disease.

All examinations were performed under anaesthesia of an intramuscular (quadriceps muscle group) injection of 3 mg/kg alfaxalone (Alfaxan CD RTU, Jurox). Once the animal was sedated, it was fully anaesthetised with 4–5% isoflurane gas and 1.5–2 L of oxygen delivered via a face mask. Sedation was maintained with 1.5–2% isoflurane gas and 1.5–2 L of oxygen for the duration of the examination [11].

### Clinical observations

The following visual and physical assessments were conducted every two weeks for urogenital, conjunctival and overall health.

Quantification of cystitis regression in koalas is currently lacking, therefore methods were developed using current clinical practice observations. Urogenital health was determined through ultra-sonographic assessment for bladder wall pathology, including thickness and presence of trabeculation (trabeculation is observed as an uneven mucosal surface within the bladder) [9, 11, 13, 20, 21]. Urinary incontinence was assessed through urine absorption at the tail stump, measured as per the Schirmers test for dry eyes [22]. Also, urine wetness of the koala rump (wet bottom), measured as wetness in millimetres from the tail stump, ascending up the koala spine [13]. Quantification of koala overall health was observed in body condition as per established methods and scored on a scale of one (emaciated) to 10 (excellent) [11]. The percent of koala dehydration, observed through skin tactility, was also recorded for overall health [11].

### Swab sample processing and DNA isolation

Urogenital (urethral for males and urogenital sinus for females), conjunctival and rectal swabs collected from koalas at each time point were swirled in either 500 μL of sterile PBS or 500 μL of RNAlater and stored at -20°C. For DNA extraction, 200 μL of the PBS homogenate from swabs was processed using the Qiagen, QIAamp DNA Mini Kit (Venlo, The Netherlands) following the “DNA Purification from Blood or Body Fluids, Spin Protocol”. All DNA aliquots were stored at -20°C until further use.

### Urogenital swab processing for RNA isolation

For total RNA extraction, 500 μL of the RNAlater homogenate from swabs was processed using the Qiagen, RNeasy Mini Kit (Venlo, The Netherlands) following the “RNA Purification from animal cells, Spin Protocol”. All isolated RNA was further treated with TURBO DNA-free (Life technologies, Carlsbad, USA) as per manufacturer instructions. Finally, all isolated RNA was precipitated in ethanol, to remove any contaminating salts. Briefly, each extract was resuspended in 50 μl of isopropanol and incubated on ice for one hour. Samples were then centrifuged at 12,000 x g for 30 min at 4°C, supernatant was discarded, and the pellet was resuspended in 500 μl of 70% ethanol. The samples were then centrifuged at 12,000 x g for 15 min at 4°C, supernatant was discarded, and the pellet was air dried at room temperature for 10 min, then resuspended in 15 μl of sterile water. All samples were then quantified for RNA determination using a Qubit RNA determination kit as per the manufacturer’s instructions. All RNA aliquots were stored at -20°C until further use.

### *C*. *pecorum* PCR detection and ompA genotyping

*C*. *pecorum* genomic DNA targeting a 209 bp fragment of the *HP* gene was PCR amplified and visualised using SYBR Green real time PCR technology [23]. Quantification was performed by plotting the crossing points against a standard curve produced using a serial dilution of known standards from 1 x 10^7^ to 1 x 10^1^ copies/μL of DNA [24].

To determine the genotype of the infecting strain, the entire *C*. *pecorum omp*A gene was amplified [25] from urogenital samples and subjected to Sanger sequencing (Macrogen, South Korea). The *C*. *pecorum ompA* genotype present was determined according to the scheme first outlined in Kollipara et al., (2013). Forward and reverse *omp*A sequences were trimmed for quality and combined into one contig using the Staden sequence analysis software. Resulting sequences were analysed by BLASTn to infer the *omp*A genotype [26].

### Total RNA sequencing

Total RNA sequencing was performed on two male and two female koala urogenital swab samples collected at baseline, week four and week eight. Isolated RNA (method described above) were sent to the University of New South Wales, Ramaciotti Centre for Genomics for library preparation and sequencing. Library preparation was performed using the SMARTer stranded total RNA-Seq kit v2—Pico input mammalian-strand-specific preparation kit. Sequencing was performed with a NextSeq 2x75 bp high output Illumina sequencer.

### Sequence quality control and mapping to the koala genome

Sequence files for each sample were concatenated into a single file and assessed for quality using the FastQC program. Each sample was mapped to the published koala annotated genome (GCF_002099425.1) using the sequence alignment program STAR (version 2.6) with default settings. Read quantification was performed using HTseq (version 0.11.2) with the options -m intersection-nonempty and -s reverse, with the subsequent count files combined in R [27]. Sequence reads deposited to SRA, BioProject identification number: PRJNA540172.

### Statistical analysis methods

All clinical measurements were assessed using a student T-test analysis for each timepoint within the graphical program ggplots (version 3.1.0), using R (version 3.5.1)[27].

Expression analysis of RNA sequencing read counts were analysed using the statistical program EdgeR (version 3.24.3) on the platform R (version 3.5.1)[27], using a quasi-likelihood approach (glmQLFit). Differential gene expression was compared between admission (week 0) to end of antibiotics (week 4) and end of antibiotics (week 4) to release (week 8).

## Results

### Antibiotic treatment improves clinical signs of cystitis in the koala

**Fig 1:**
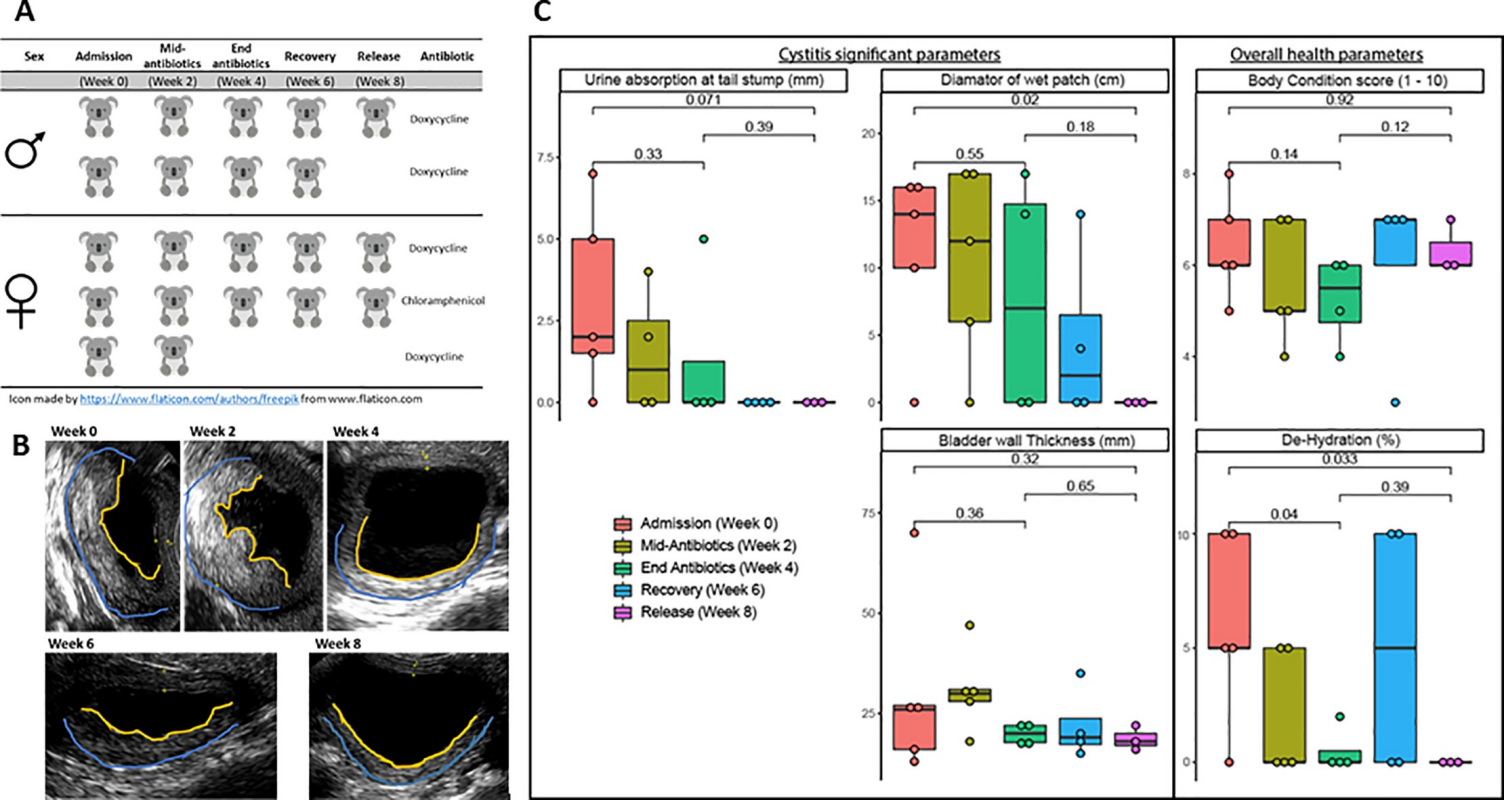
**Experimental design (A) and cystitis regression markers (B and C) in response to antibiotic treatment over the eight week trial period.** (A)Experimental design, included five koalas (two male and three female), with clinical cystitis. Koalas were examined at admission (week 0), mid-antibiotic treatment (week 2), end of antibiotic treatment (week 4), two weeks into recovery (week 6) and at release (week 8). (B) Ultra-sonographic images of the bladder wall from a male koala at each time point, showing resolution of bladder wall trabeculation (smoothing of the bladder wall mucosal surface, observed in the yellow line) and thinning of the bladder wall observed in the distance between uroepithelium (yellow line) and the lamina propria (blue line). (C) Clinical measurements for both cystitis parameters and overall health parameters. Observations of cystitis indicate significant decrease in urinary incontinence and trends of bladder wall thinning. Observations of overall health indicate significant improvement in percentage dehydration but not body condition. Student T-test was used to calculate statistical significance.

Quantification of cystitis resolution was observed at two weekly intervals through the monitoring of five koalas (two male and three female) with clinically diagnosed cystitis over a period of eight weeks (Fig 1A). Specific markers of disease regression were measured at three different sites; (1) bladder wall (2) tail stump (3) rump (wet bottom) (Fig 1B and 1C). At admission to the wildlife hospital, all koalas had signs of bladder wall thickening and the presence of trabeculation. Urinary incontinence was observed in all koalas, with an average of 3.1 mm of urine absorption on a Schirmers tear test strip and an average of 11.2 cm urine spread in the surrounding fur. After the initial two weeks of rest, antibiotic treatment and subcutaneous fluids (administered when needed, post assessment of skin tactility), bladder wall thickness showed no improvement, with a slight increase in wall thickness by 0.4 mm (from 30.4 mm to 30.8 mm). However, there was a decrease in signs of urinary incontinence by both urine absorption (from 3.1 mm to 1.2 mm) and urine spread in the surrounding fur (from 13.2 mm to 10.4 mm) (all measurements recorded in S1 Table and S1 Fig). By the completion of antibiotic treatment (week four), all three clinical parameters showed improvement and by week eight, there was a significant decrease in urine absorption (3.1 mm to 0 mm; p, 0.071) and urine spread in the surrounding fur (14 mm to 0 mm; p, 0.02) as compared to admission (Fig 1C). At week eight, all signs of wet bottom were absent, trabeculation had resolved and the thickness of the bladder wall had decreased by an average of 38.5% (30.4 mm to 18.7 mm) compared to admission (Fig 1B). It was also noted that a single female koala had concurrent conjunctival pathology related to chlamydial infection. This disease presentation also resolved within the treatment period.

The overall health of each koala was also monitored through body score condition (muscle mass over the scapular) and dehydration (skin tactility). At admission, all koalas had a moderate body score (5–8 on a 10-point scale). Over the course of treatment, this condition remained constant and is thought to be a result of both the antibiotics (halting disease progression) and the koala’s restricted movement during the treatment period (limiting muscle development). Observations of koala dehydration resolution were significant within the antibiotic treatment period (5% to 0%; p, 0.04) and over the trial period (5% to 0%; p, 0.033). However, there were observations of increased dehydration during the recovery period (week 6) (Fig 1C) (S1 Table). Also of note, unfortunately, two of the five koalas did not finish the trial and had to be euthanised. A female koala was euthanized at week two of the trial due to advanced reproductive and urinary tract disease, while a male koala was euthanised at week six of the trial due to gastrointestinal complications, resulting in muscle wasting and dehydration. Despite this male koala’s adverse outcome from treatment complications, he did resolve his signs of cystitis before being euthanised.

### Antibiotic treatment results in removal of chlamydial DNA, as measured by qPCR, in all koalas

**Fig 2:**
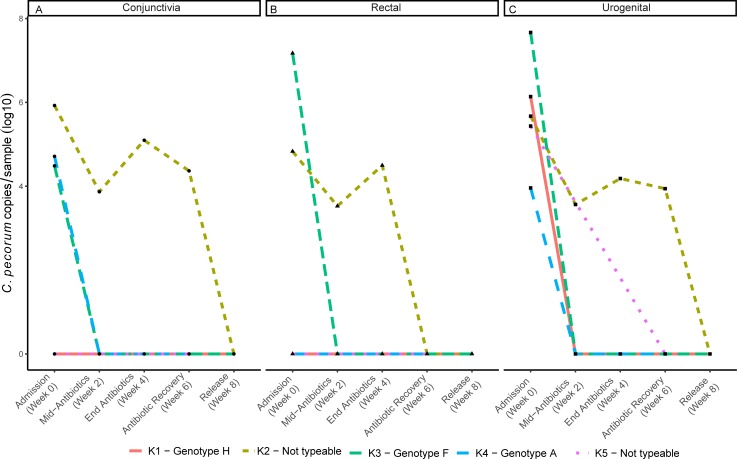
*C*. *pecorum* load detected by qPCR in trial koalas. *C*. *pecorum-*specific DNA was monitored at (A) conjunctival, (B) rectal and (C) urogenital tract sites throughout the trial. Corresponding *C*. *pecorum ompA* genotypes associated with each koala are listed at the bottom.

*C*. *pecorum* DNA load was assessed using a specific qPCR assay to determine bacterial DNA loads at conjunctival (Fig 2A), rectal (Fig 2B) and urogenital (Fig 2C) sites at 0, 2, 4, 6 and 8 weeks post admission. Three of the five koalas had chlamydial DNA clearance by week 2, with the fourth koala clearing its chlamydial DNA by week 6 and the final koala clearing its chlamydial DNA by week 8 of the trial (Fig 2) (S1 Table). The strain of the infecting *C*. *pecorum* was determined using the *ompA* genotype at the urogenital site. For the three koalas where genotyping was successful, each koala was found to be infected with a different genotype (H, F and A).

### Identification of 468 differentially expressed genes at the uroepithelium site

**Fig 3:**
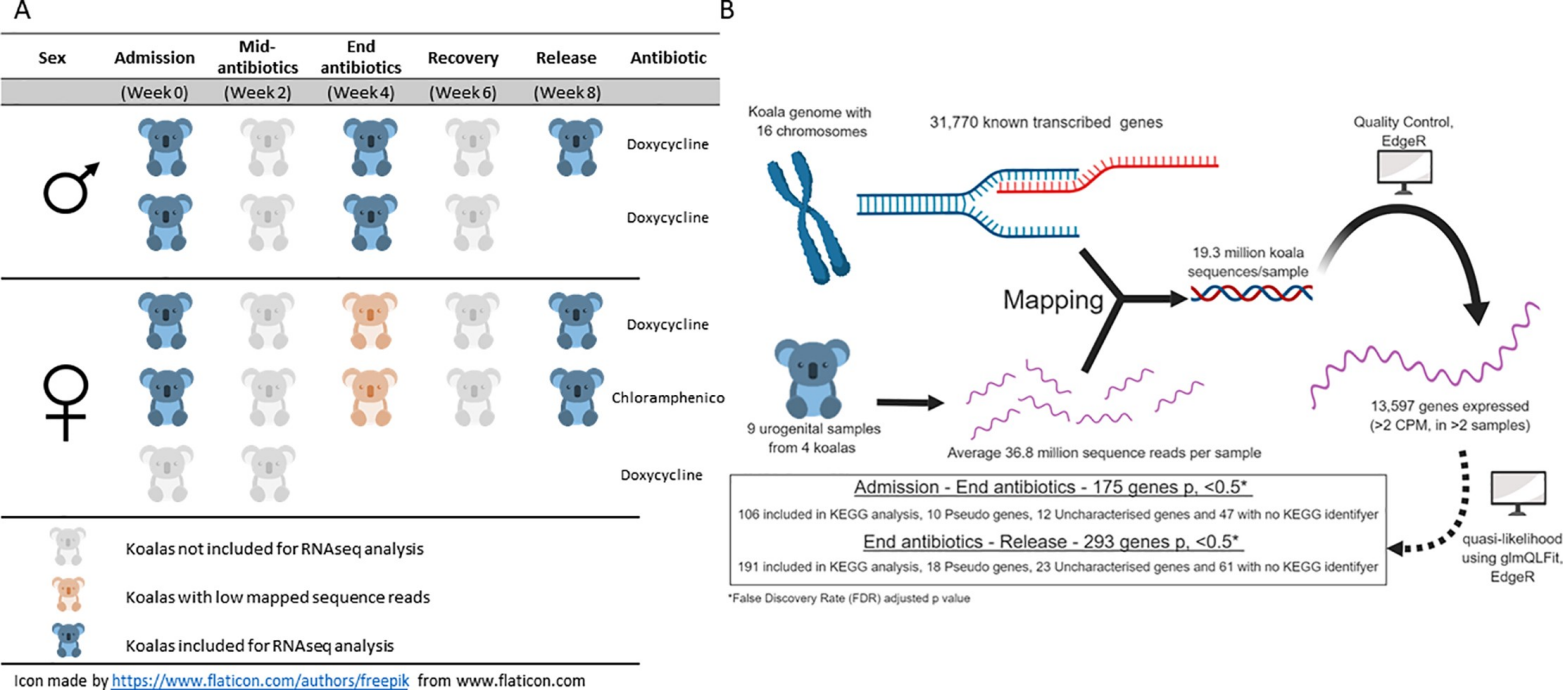
Transcriptomic design, bioinformatic quality control and filtering of expressed genes. (A) Experimental design, included four koalas (two male and two female) with clinical cystitis. Samples assessed were from admission (week 0), end of antibiotic treatment (week 4) and at release (week 8). (B) Bioinformatic analysis of sequences, including mapping to the koala reference genome, quality control (>2 counts per million (CPM) in >2 samples), quasi-likelihood statistical analysis (using glmQLFit) approach and filtering to differentially expressed genes (adjusted p < 0.5).

Gene expression at the site of disease was assessed using RNA sequencing analysis of urogenital swabs from three koalas (one male and two female) at 0, 4 and 8 weeks, and an additional male koala at 0 and 4 weeks only. These 11 samples represent the following time points during treatment; 1) Upon admission to the wildlife hospital, representing the gene expression profile of a koala with unresolved chlamydial-induced cystitis (Week 0); 2) Post antibiotic therapy, representing the gene expression profile in response to antibiotic treatment for 4 weeks (Week 4); 3) Before release, representing the gene expression profile of a koala recently recovered from chlamydial-induced cystitis (Week 8) (Fig 3A). Total RNA sequencing obtained an average of 36.8 million reads per sample, of which an average of 19.3 million sequence reads per sample mapped to the koala reference genome. Of the 11 data sets from four koalas, two samples (both female week 4 samples) failed quality control (mapped library size of 321,662 and 444,088 sequence reads) so they were subsequently removed from further analysis (Fig 3A).

The koala genome consists of over three billon base pairs coding over 31,000 genes, on 16 chromosomes [28]. Expression of these genes within the current dataset was analysed using the statistical program EdgeR (version 3.24.3) on the platform R (version 3.5.1) [27]. This identified a total of 13,597 genes expressed with a minimum of two counts per million (CPM) over a minimum of two samples (Fig 3B). Due to high biological variation between samples (the koalas used in the study are all outbred), a higher than normal false discovery rate (FDR) was used to identify differentially expressed genes in this dataset. Of the 13,597 expressed genes, 175 were observed as differentially expressed (adjusted p, < 0.5) from admission (week 0) to the end of antibiotic treatment (week 4). A further 293 genes were observed as differentially expressed (adjusted p, < 0.5) from the end of antibiotic treatment (week 4) to release (week 8) (Fig 3B) (S2 Table).

### KEGG Pathway similarities and differences between comparison groups

KEGG pathway analysis of the genes differentially expressed across both time periods identified 21 key pathways with changes in gene expression during the trial period (pathways involving ≥3 genes) [29, 30]. Five of these pathways involved genes differentially expressed across the entire trial period (admission to release), while eight pathways involved genes differentially expressed between admission (week 0) and the end of antibiotic treatment (week 4) and another eight separate pathways involved genes differentially expressed between the end of antibiotic treatment (week 4) and release (week 8) (Table 1).

**Table 1:** Pathways involving ≥3 differentially expressed genes between the antibiotic treatment period (Week 0 to Week 4) and the recovery period (Week 4 –Week 8).

Pathway	*Admission (Week 0) to End of Antibiotic treatment (Week 4) Genes*	*End of Antibiotic treatment (Week 4) to Release (Week 8) Genes*
Metabolic Pathways	*PLCD*, *CYP2B*, *B3GNT4*	*AOX*, *GALNT*, *dgkA*, *A4GALT*, *IL4I1*, *PLCB*, *B3GNT4*, *RRM2*, *PIP5KL1*
Phospholipase D signalling	*CXCR2*, *FCER1G*, *IL8*	*dgkA*, *PDGFRB*, *PLCB*, *FCER1G*, *IL8*
Calcium signalling	*CCKBR*, *ADORA2B*, *CAMK2*, *PLCD*	*CACNA1C*, *CACNA1I*, *PDGFRB*, *GRIN2C*, *PLCB*
Cytokine-cytokine receptor interaction	*IL1β*, *AMH*, *CXCR2*, *CSF3R*, *IL1RN*, *IL8*	*AMH*, *CSF3R*, *CCL3*, *IL8*, *CCL4*
Neuroactive ligand-receptor interaction	*FPRL*, *CCKBR*, *ADORA2B*, *GRIN2B*, *P2RY13*	*OPRL1*, *PTGER2*, *CXCR8*, *CHRNA2*, *NMUR1*, *GRIN2C*, *P2RY8*, *UCN*
Necroptosis	*CAMK2*, *IL1β*, *NOX2*	N/A
Axon guidance	*CAMK2*, *SEMA3*, *SRGAP*	N/A
Osteoclast differentiation	*IL1β*, *SOCS3*, *NCF1*, *SPI1*	N/A
NOD-like receptor signalling	*IL1β*, *IL8*, *MEFV*, *Bf-CRAMP*, *NOX2*	N/A
C-type lectin receptor signalling	*IL1β*, *FCER1G*, *CLEC4E*	N/A
Hematopoietic cell lineage	*IL1β*, *CSF3R*, *CD33*	N/A
cAMP signalling	*CAMK2*, *AMH*, *GRIN2B*, *HCAR2*	N/A
Chemokine signalling	*CXCL2*, *NCF1*, *IL8*	N/A
MAPK signalling	N/A	*RASGRF1*, *CACNA1C*, *CACNA1I*, *PDGFRB*, *FLT1*, *FLT4*
Ras signalling	N/A	*RASGRF1*, *PDGFRB*, *FLT1*, *FLT4*, *RGL1*
Phagosome	N/A	*THBS2S*, *CD36*, *COLEC12*, *MARCO*, *NOX2*
PI3K-Akt signalling	N/A	*THBS2S*, *CSF3R*, *PDGFRB*, *FLT1*, *FLT4*, *COL1A*, *COL4A*, *COL6A*
Focal adhesion	N/A	*RASGRF1*, *THBS2S*, *PDGFRB*, *FLT1*, *FLT4*, *COL1A*, *COL4A*, *COL6A*
ECM-receptor interaction	N/A	*THBS2S*, *COL1A*, *COL4A*, *COL6A*, *HSPG2*, *CD36*
Circadian entrainment	N/A	*CACNA1C*, *CACNA1I*, *GRIN2C*, *PLCB*, *GUCY1A*
Glutamatergic synapse	N/A	*CACNA1C*, *GRIN2C*, *SLC1A3*, *PLCB*, *SHANK*

Down-regulated, Up-regulated

During the entire study period, however, the expressed genes within these pathways are distinct between the antibiotic period and the recovery period. Finally, pathways specific to the recovery period were also identified that appear to be a response to cellular proliferation and differentiation.

Eight of the pathways regulated between admission (week 0) and the end of antibiotic treatment (week 4) involve the regulation of interleukins (*IL*) *IL1β* and/or *IL8*. The NOD-like receptor signalling pathway includes the regulation of both these interleukins and is directly connected to other identified pathways including cytokine-cytokine receptor interactions, calcium signalling pathways, C-type lectin receptor signalling pathways and chemokine signalling pathways. Other commonalities between the identified pathways in this group include common processes such as cAMP signalling pathways, metabolic pathways, neuroactive ligand receptor interactions and the phospholipase D signalling pathway. Of the pathways regulated between the end of antibiotic treatment (week 4) and release (week 8), there are similarities and differences when compared to the first half of the study (admission to the end of antibiotic treatment). The pathways involved in inflammation are either absent or involved different differentially expressed genes after antibiotic treatment finished, compared to during antibiotic treatment. During recovery, there is a complete absence of NOD-like receptor and chemokine signalling gene expression and a major shift in the profile of cytokine-cytokine receptor interaction gene expression, with genes *IL8* and *CSF3R* changing from being down-regulated to up-regulated. In addition, the gene *AMH* changed from being down-regulated during antibiotic treatment to up-regulated during recovery. Pathways involved in fundamental cellular process including metabolic pathways, neuroactive ligand receptor interactions and phospholipase D signalling had significant gene expression.

### Inflammation regulated through chemokine signalling pathways involving *NCF1* and *NOX2* resulting in the down-regulation of genes involved in pathways leading to reactive oxygen species

**Fig 4:**
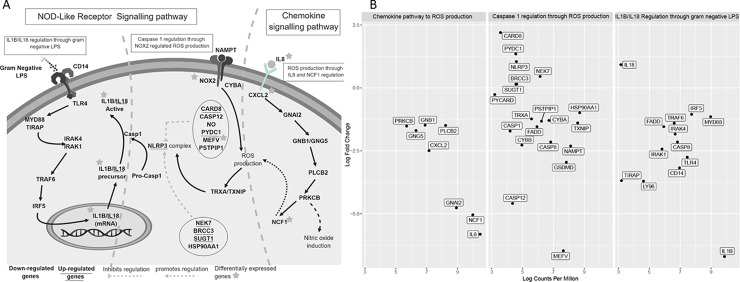
Gene expression changes between admission (week 0) and the end of antibiotic treatment (week 4) involved in *IL1β/IL18* transcription and activation. Key pathways were identified based on significant genes from Table 1. (A) Pathway cascades involved in three key pathways identified in Table 1, indicating the likely activation of caspase 1, *IL1β* and *IL18*. (Up-regulated genes are underlined and genes identified in Table 1 are indicated with a star). (B) Log fold change and log counts per million (CPM) of each gene involved in the transcription and activation of *IL1β/IL18*, separated into three pathway sub-sections; 1) Chemokine pathway to reactive oxygen species (ROS) production. 2) Caspase 1 regulation through ROS production. 3) *IL1β/IL18* regulation through gram negative *LPS* detection.

The genes *NCF1* and *NOX2*, which are involved in the NOD-like receptor interaction pathway, cytokine-cytokine signalling and chemokine signalling (Table 1) and were identified as significantly down-regulated during antibiotic treatment (Fig 4A).

Analysis of the genes upstream of *NCF1* regulation of reactive oxygen species (ROS) production, in the chemokine receptor pathway, identified that all six genes (*PRKCB*, *GNB1*, *PLCB2*, *GNG5*, *CXCL2* and *GNAI2*) between *IL8* and *NCF1* were down-regulated during antibiotic treatment, with *IL8* and *CXCL2* also identified as significant (Table 1) (Fig 4A and 4B).

Furthermore, analysis of genes downstream of ROS production, leading to control of the *NLRP3* inflammasome complex and caspase 1 (in other hosts), also showed a trend of down-regulation (6 genes up-regulated and 14 genes down-regulated) with *MEFV* also identified as significant (Table 1) (Fig 4A and 4B). Caspase 1 activation is well known in other hosts for its activation of the pro-inflammatory cytokine *IL1β*, a cytokine also identified as significantly down-regulated during antibiotic treatment in our koalas. *IL1β* transcription is commonly stimulated through *LPS* detection and receptors *CD14* and *TLR4* (in other hosts), which also had the trend of down-regulation during antibiotic treatment in our koalas (1 gene up-regulated and 12 genes down-regulated) (Fig 4A and 4B).

## Discussion

In the current study, an extensive clinical protocol was used to define and quantify cystitis regression in koalas with current chlamydial infections of the uroepithelium during a course of antibiotic treatment (doxycycline or chloramphenicol), subcutaneous fluids and supportive care.

Over the course of antibiotic treatment (4 weeks), there was significant disease regression. This was measured by the resolution of trabeculation, thinning of the bladder wall, complete regression of urinary incontinence and clearance of chlamydial DNA from all mucosal sites. Despite the improvement in cystitis signs, overall health indicators for koalas did not improve significantly. Current methods for determining koala health are set out in two key resources: “Medicine of Australian mammals” and “Koala Rehabilitation Manual” [11, 13]. These resources indicate that a koala’s skin tactility and muscle mass over the scapular are reliable indicators of hydration status and body condition, respectively [11, 13]. However, limited improvements in these indicators were noted throughout the trial, with minor improvements in koala hydration status and no change in muscle mass observations.

While the number of animals analysed was very small, the preliminary data showed that strains of *C*. *pecorum* (as defined by ompA genotype) did not directly relate to treatment outcomes.

Studies on *Chlamydia caviae* infections in the guinea pig urethra report a marked inflammatory immune response without the development of fibrosis or scarring [5, 6]. Interestingly, unlike observations seen in the koala, ascension of *Chlamydia* to the bladder and development of cystitis appears to be rare in immunologically intact hosts [6]. Previous reports of urogenital pathology in koalas have had limited success in quantifying the disease state and none have followed disease regression through antibiotic treatment. Patterson et al. (2015) reported the most detailed information of urogenital disease within Victorian koalas. They discuss the possibility of disease severity reflecting differences in causative agents, reporting a relationship with *Chlamydia* positivity to increases in disease severity [9]. However, there are no reports of how these koalas responded to treatment and the study lacked quantitative analysis from ultra-sonographic imaging [9]. Our current study is the first to quantify and follow cystitis disease regression in koalas during antibiotic treatment and onward through recovery to release.

A transcriptomic approach was adopted to uncover the immunological responses present during chlamydial clearance and disease regression in the koala. This revealed evidence that the uroepithelium goes through two major transcriptomic changes during resolution of chlamydial-induced cystitis: one related to inflammation (week 0 –week 4) and one related to healing (week 4 –week 8). The koala immune profile during antibiotic treatment indicates a significant reduction in pro-inflammatory pathways including NOD-like receptor signalling, cytokine-cytokine receptor interactions, calcium signalling, C-type lectin receptor signalling and chemokine signalling. The common cytokines highlighted in these pathways are *IL1β* and *IL8*. In other hosts, *IL1β* has been shown to be a tightly regulated cytokine, requiring stimulation of caspase 1 activation pathways through the *NLRP3* inflammasome, to cleave *pro-IL1β* into its active form [31, 32].

Reports of *IL1β* involvement in chlamydial infections have been demonstrated in both *Chlamydia pneumoniae* and *Chlamydia muridarum* mouse respiratory infections. He *et al*. (2010) demonstrated that *C*. *pneumoniae* has a greater potential than *C*. *muridarum* to induce *IL1β* from bone marrow derived macrophages during lung infections in mice. They also demonstrated a reduction in pathology and neutrophil involvement in *IL1*-defective mice during *C*. *pneumoniae* infections [33]. Conversely, a report in 2011 indicated that *IL1β* and caspase 1 were required for effective *C*. *pneumoniae* clearance in mice, reporting observations of increased infections and pathology in caspase 1-defective mice [34]. Also noted by Shimada *et al*. (2011) was a lack of ROS involvement in the activation of the *NLRP3* inflammasome complex. In our current koala *Chlamydia* study, significant down-regulation of *IL1β* was observed during antibiotic treatment and coincided with the clearance of chlamydial shedding and regression in the signs of chlamydial-induced cystitis.

Analysing specific genes involved in the down-regulation of the inflammatory immune response, koalas with chlamydial-induced cystitis provided evidence supporting a role for ROS production. *IL8*, *CXCL2* and *NCF1* were all observed to be significantly down-regulated during antibiotic treatment. These three genes are known to be involved in a specific pathway resulting in ROS production [35]. Furthermore, ROS production is also known to be directly regulated through *NOX2* [36], which was also identified as a significantly down-regulated gene during antibiotic treatment. Combined, the significant down-regulation of both *NCF1* and *NOX2* would lead to a decrease in ROS production.

Further studies in tissues other than the uroepithelium have also demonstrated that increased ROS production results in increased *Chlamydia trachomatis* infection loads. Furthermore, inhibition of the ROS production decreased chlamydial loads [37]. This supports our current findings with decreased ROS production (observed through *NCF1* and *NOX1* down-regulation) coinciding with *C*. *pecorum* clearance.

ROS production through *NCF1* has been demonstrated in other intracellular bacteria previously. *NCF1*-defective mice infected with *Mycobacterium marinum* demonstrated increased infection loads compared to wild type mice, which also resulted in increased *IL1β* activity [38]. However, when macrophages and neutrophils were observed separately, contrasting effects were seen. *NCF1*-defective mice had macrophages with decreased levels of *IL1β* and neutrophils with increased levels of *IL1β*. The authors concluded that *IL1β* induced in macrophages through ROS production was controlled by *NCF1*, whereas *IL1β* induced in neutrophils through ROS production was independent of *NCF1* (38). This information suggests that, in the current dataset, the two separate pathways identified leading to ROS production (chemokine and NOD-like receptor signalling pathways) may be due to separate cell types. However, single cell RNA sequencing data will be needed to confirm this hypothesis.

Pathways not related to inflammation, but also regulated during the antibiotic treatment period (week 0 to week 4) in our koalas, indicated fundamental changes in cellular process including regulation of cAMP signalling pathways, metabolic pathways, neuroactive ligand receptor interactions and phospholipase D signalling pathway. Specific functions for the regulation in these pathways were difficult to interpret, however these pathways are involved in cellular healing processes and clean-up of cellular debris that would be present after bacterial clearance and down-regulation of the inflammatory immune response. Further studies are required to determine the exact mechanisms involved.

Regulation of koala immune pathways during the recovery period (week 4 –week 8) indicates significant down-regulation in cellular proliferation and differentiation pathways, including ECM-receptor interactions, focal adhesion pathways and PI3K-Akt signalling pathways. There was also a notable absence of inflammatory pathways that were indicated during the antibiotic treatment period. Cellular proliferation and differentiation suggests possible continued tissue healing and remodelling. Cellular healing, specifically in uroepithelium tissue due to bacterial infections, is poorly understood and limits the conclusions that can be made from these findings. However, reports from mechanical injury to the bladder wall have been studied extensively in other hosts and indicate a complex and continuous process [39]. This process has been defined into three separate stages; 1) inflammation, 2) new tissue formation and 3) remodelling [40–42]. In the current koala *Chlamydia* study, significant down-regulation of several genes involved in collagen formation (*COL1A*, *COL4A* and *COL6A*) and endothelial growth (*FLT1* and *FLT4*) were observed at the end of treatment. Collagen formation and endothelial growth are involved in migration and proliferation healing processes and, in other hosts, and have been shown to be rapidly (within 12 hours) up-regulated in response to epithelial damage [43, 44]. In our study, the absence of any change (up or down-regulation) in these processes until the final treatment period (week 4—week 8) also indicates that tissue healing was already present at the time of admission and lasted for a period of between four and eight weeks post admission.

This study has acknowledged limitations, including the inherent nature of RNA sequencing of mixed cellular types and the small number of outbred koalas included in the trial. Without differentiation of specific cell types within each sample and a large cohort of replicate samples for each time point, the expression profiles between samples contained a range of variability. This resulted in increased biological variation and a decreased sensitivity of detecting differentially expressed genes. Further limitations include the intermittent use of steroidal injections, which can affect the immunological immune responses observed. Nonetheless, key genes involved in pathways related to reactive oxygen species production and tissue healing were identified by comparing differentially expressed genes between admission, end of antibiotic treatment and recovery periods. This study provides a first-glimpse at the pathways involved chlamydial-induced cystitis inflammation and healing. Future studies focusing on these genes in controlled trials will provide important evidence towards the mechanisms of chlamydial clearance and cystitis regression within the koala proposed here.

In conclusion, the present study demonstrates that treating koalas with current *C*. *pecorum* infections and signs of cystitis with antibiotics results in chlamydial clearance and disease regression through the down-regulation of 21 genetic pathways. Furthermore, data indicated that the pathways leading to ROS production was down-regulated via two separate pathways involving *NCF1* (via *IL8*) and *NOX2*, which have possible downstream effects on caspase 1 and *IL1β* activation. Using these results, future trials studying the therapeutic effects of experimental vaccines may be able to accurately monitor shifts in innate immune responses, possibly before clinical representations are apparent. It also indicates possible immune pathways involved in chronic chlamydial infections at mucosal sites, not only in koalas, but other hosts and chlamydial species.

### Animal ethics approvals

Ethical approval for this study was granted by University of the Sunshine Coast, Animal Ethics Committee (AEC CA No. AN/S/17/49) and was performed under a Queensland Scientific Purposes Permit granted by Queensland Government, Department of Environment and Heritage Protection (SPP No. WA0001189).

## Supporting information

S1 Fig Bladder wall images of each koala at each time point taken using ultrasonography technology. (PDF)

S1 Table Measurements for all clinical data and *C*. *pecorum* PCR quantification results for all sites and time points. (PDF)

S2 Table Significantly differentiated genes identified using EdgeR quasi-likelihood (using glmQLFit) for both treatment periods (antibiotic treatment and recovery). (PDF)
