# Supplementary material for: Antibiotic treatment of Chlamydia-induced cystitis in the koala is linked to expression of key inflammatory genes in reactive oxygen pathways
Source: PLoS One. 2019 Aug 15;14(8):e0221109. doi: 10.1371/journal.pone.0221109 (PMC6695219; doi:10.1371/journal.pone.0221109)
Supplement: S1 Fig — (PDF) [file pone.0221109.s001.pdf]

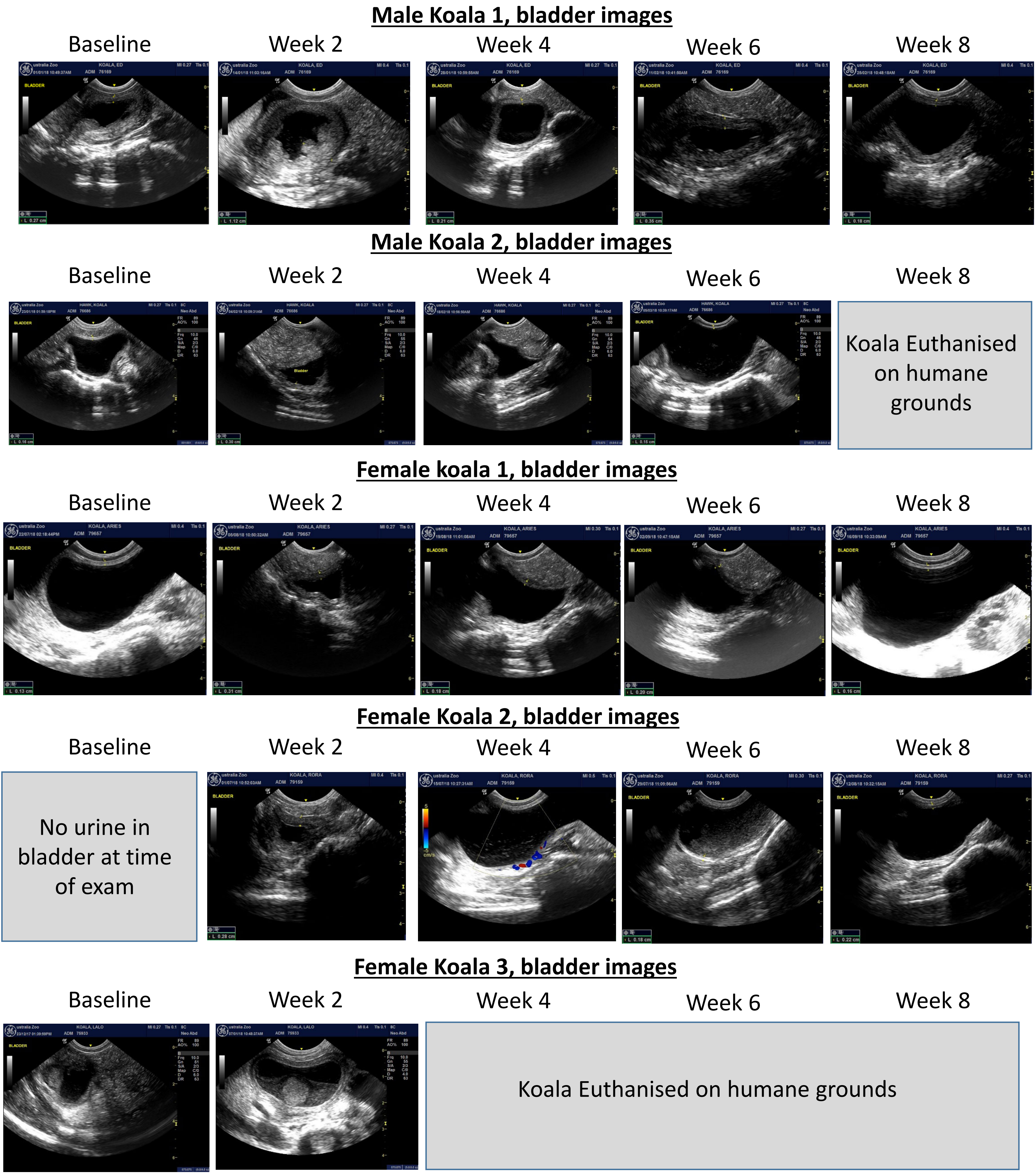

Supplementary figure 1. Ultrasound images of the bladder for each koala at every time point
