# Supplementary material for: Antibiotic treatment of Chlamydia-induced cystitis in the koala is linked to expression of key inflammatory genes in reactive oxygen pathways
Source: PLoS One. 2019 Aug 15;14(8):e0221109. doi: 10.1371/journal.pone.0221109 (PMC6695219; doi:10.1371/journal.pone.0221109)
Supplement: S1 Table — (PDF) [file pone.0221109.s002.pdf]

Supplementary table 1. Clinical and molecular measurments for all koalas at all avalible time points

Test	Week	Urine absorbance at tail stump (mm)	Diamator of wet patch (cm)	Bladder wall Thickness (mm)	De-hydration (%)	Body Condition Score (1 -10)	C. pecorum - Ocular (copies/swab)	C. pecorum - Rectal (copies/swab)	C. pecorum - Urogenital (copies/swab)
Male Koala 1 (Doxycycline)	Baseline	1.5	14	27	0	6	0	0	1.37E+06
	Week 2	4	17	47	0	5	0	0	0
	Week 4	0	14	22	0	5	0	0	0
	Week 6	0	14	35	0	7	0	0	0
	Week 8	0	0	18	0	6	0	0	0
Male koala 2 (Doxycycline)	Baseline	2	16	16	5	5	5.16E+04	0	9.12E+03
	Week 2	2	12	30	0	5	0	0	0
	Week 4	5	17	22	2	4	0	0	0
	Week 6	0	4	15	10	3	0	0	0
	Week 8	N/A	N/A	N/A	N/A	N/A	N/A	N/A	N/A
Female koala 1 (Doxycycline)	Baseline	7	10	26	10	7	8.43E+05	6.78E+04	4.70E+05
	Week 2	0	6	28	0	7	2.33E+04	0	8.75E+03
	Week 4	0	0	17	0	6	1.25E+05	3.13E+04	1.53E+04
	Week 6	0	0	18	0	7	7.37E+03	3.36E+03	3.67E+03
	Week 8	0	0	22	0	7	0	0	0
Female koala 2 (Chloramphenicol)	Baseline	0	10	13	5	8	3.07E+04	1.49E+07	4.65E+07
	Week 2	0	0	31	5	7	0	0	0
	Week 4	0	0	18	0	6	0	0	0
	Week 6	0	0	20	10	7	0	0	0
	Week 8	0	0	16	0	6	0	0	0
Female koala 3 (Doxycycline)	Baseline	5	16	70	10	6	0	0	2.72E+05
	Week 2	Not taken	17	18	5	4	0	0	0
	Week 4	N/A	N/A	N/A	N/A	N/A	N/A	N/A	N/A
	Week 6	N/A	N/A	N/A	N/A	N/A	N/A	N/A	N/A
	Week 8	N/A	N/A	N/A	N/A	N/A	N/A	N/A	N/A
