# Supplementary material for: Antibiotic treatment of Chlamydia-induced cystitis in the koala is linked to expression of key inflammatory genes in reactive oxygen pathways
Source: PLoS One. 2019 Aug 15;14(8):e0221109. doi: 10.1371/journal.pone.0221109 (PMC6695219; doi:10.1371/journal.pone.0221109)
Supplement: S2 Table — (PDF) [file pone.0221109.s003.pdf]

Supplementary table 2. Differentially expressed genes between each time point with corresponding kegg number (KO#), log fold change (logFC), log count per million (logCPM), F value (F), P value (PValue), false discovery rate adjusted P value (FDR), Predicted protein (function) and the koala refrence genome location number (LOC#)

Admission (week 0) to End of Antibiotics (Week 4)

genes	KO#	logFC	logCPM	F	PValue	FDR	function	LOC#
LOXL4	K00280	6.560337	4.344205	7.906544	0.004927	0.437841		
FMO6P	K00485	9.559361	2.506135	7.601086	0.005835	0.469427	putative di	LOC11019
CHST11	K01017	8.904431	4.109732	10.82447	0.001002	0.271327		
RNASE6	K01172	-11.474	4.791521	7.926453	0.004873	0.437504	ribonucleas	LOC11020
PGLYRP2	K01446	-6.17224	8.165478	9.8775	0.001674	0.350116		
FPR2	K04173	-11.8428	5.107725	9.11842	0.002531	0.37908	formyl pep	LOC11021
CCKBR	K04195	10.9645	3.385968	10.75991	0.001038	0.271327		
ADORA2B	K04267	-11.7749	5.132563	9.099536	0.002558	0.37908		
MAPT	K04380	8.217309	4.004936	9.333425	0.002251	0.37908		
CAMK2B	K04515	5.759141	4.799363	7.678084	0.005591	0.467981		
IL1B	K04519	-6.70047	9.867449	12.23797	0.000469	0.213505		
ADGRE1	K04591	-5.69382	8.742069	9.267824	0.002333	0.37908		
ADGRB3	K04598	8.960652	4.518575	13.01208	0.00031	0.168425		
AMH	K04665	9.344645	3.319405	8.655523	0.003262	0.385212		
SOCS3	K04696	-5.04184	9.672381	8.218499	0.004148	0.409656		
IRAK2	K04731	-5.45693	8.376339	8.569838	0.003419	0.385212		
CHST12	K04742	5.554003	5.126201	7.978463	0.004735	0.437504		
TRPV5	K04974	10.04719	2.644449	8.258624	0.004057	0.407767		
KCNJ12	K05005	7.980121	3.95335	8.815561	0.002988	0.383229	ATP-sensiti	LOC11022
CXCR2	K05050	-5.89779	7.197474	8.467994	0.003615	0.390156		
CSF3R	K05061	-6.78302	11.15352	13.23992	0.000274	0.162111		
GRIN2B	K05210	8.787381	4.733966	13.44169	0.000246	0.158879		
NRG3	K05457	10.27636	2.711327	8.578579	0.003402	0.385212		
IL1RN	K05481	-5.91344	10.18357	10.54362	0.001166	0.299228		
TGM1	K05619	-5.40294	8.067633	8.212708	0.004161	0.409656		
VIL1	K05761	7.710396	3.950035	8.409853	0.003733	0.393462		
PLCD4	K05857	5.718047	4.891227	7.825351	0.005153	0.44628		
SPTBN5	K06115	5.474314	5.494331	8.950701	0.002774	0.381054		
CD33	K06473	-5.64459	8.009557	8.651667	0.003269	0.385212	myeloid ce	LOC11022
SPN	K06477	-5.62813	8.711057	9.075118	0.002592	0.37908		
NECTIN2	K06531	-5.16626	7.908332	7.628121	0.005748	0.467981	nectin-2	LOC11021
TMEM233	K06566	-6.15284	7.415103	9.074478	0.002593	0.37908	dispanin su	LOC11020
IFITM2	K06566	-6.81617	7.84318	10.9367	0.000943	0.271327	interferon-	LOC11020
VCAN	K06793	-5.64795	11.5588	10.50339	0.001192	0.30015		
SEMA3D	K06840	6.856967	4.372712	8.523796	0.003506	0.385212		
SRGN	K06849	-5.4661	10.0283	9.379717	0.002195	0.37908		
CYP2B11	K07412	10.51727	2.827089	8.978343	0.002733	0.379163	cytochrom	LOC11019
SRGAP3	K07526	9.819184	4.61679	14.9211	0.000112	0.127119		
KRT85	K07605	10.35428	2.612397	8.56467	0.003428	0.385212	keratin, typ	LOC11019
B3GNT4	K07971	9.109081	3.197174	8.075417	0.004488	0.419449		

End of Antibiotics (Week 4) to Release (Week 8)

genes	KO#	logFC	logCPM	F	PValue	FDR	function	LOC#
AOX1	K00157	-10.5916	3.210946	7.680883	0.005582	0.419336		
LOXL4	K00280	-12.0633	4.344205	12.91232	0.000327	0.169623		
GALNT9	K00710	-10.5919	3.038824	7.36872	0.006638	0.442037		
DGKB	K00901	-11.4127	3.746671	9.747462	0.001796	0.313216		
LPL	K01059	-10.8502	3.517634	8.567023	0.003424	0.385571		
INPP5J	K01106	-10.442	3.22727	7.572204	0.005929	0.419336		
C2	K01332	-10.2738	4.199803	9.711218	0.001832	0.313216		
PGLYRP2	K01446	5.415421	8.165478	8.021899	0.004623	0.406923		
AZIN2	K01583	-7.68087	5.082959	9.456211	0.002105	0.327986	antizyme ir	LOC110206237
A4GALT	K01988	-6.95796	4.663959	6.644289	0.009949	0.488371		
IL4I1	K03334	-5.4257	5.55691	6.978822	0.00825	0.465433		
SKP2	K03875	-10.5159	2.896064	7.069128	0.007844	0.451199		
A2M	K03910	-10.2038	2.767364	6.692241	0.009685	0.482388		
OPRL1	K04216	-11.0601	3.557328	8.873571	0.002894	0.371241		
PTGER2	K04259	-10.4415	3.446012	8.00102	0.004676	0.406923		
gpr35	K04276	-5.28902	5.517227	6.622234	0.010073	0.489235	G-protein c	LOC110198099
RASGRF1	K04349	-11.0806	3.488664	8.735097	0.003122	0.378659		
ADGRB3	K04598	-12.2596	4.518575	14.05638	0.000178	0.120696		
THBS2	K04659	-11.3636	3.747881	9.697218	0.001846	0.313216		
AMH	K04665	-11.0636	3.319405	8.340876	0.003877	0.399392		
SOCS3	K04696	5.042751	9.672381	8.021253	0.004624	0.406923		
CHRNA2	K04804	-6.99967	5.954603	9.960955	0.0016	0.31032		
CACNA1C	K04850	-5.11671	5.612054	6.59043	0.010255	0.489235		
CACNA1I	K04856	-9.11419	3.96535	7.747774	0.005379	0.419336		
KCNK13	K04922	-11.3965	3.744749	9.723571	0.00182	0.313216		
KCNN1	K04942	-10.6468	3.410949	8.131028	0.004352	0.406923		
KCNJ12	K05005	-11.692	3.95335	10.77001	0.001032	0.268071	ATP-sensiti	LOC110223210
CLIC4	K05024	6.745639	6.400656	8.346434	0.003865	0.399392		
ABCC8	K05032	-10.4736	3.032401	7.253436	0.007078	0.442037		
SLC6A17	K05048	-8.948	4.240739	8.491492	0.003569	0.385571		
NMUR1	K05052	-10.8373	3.10633	7.710571	0.005491	0.419336		
CSF3R	K05061	5.537437	11.15352	9.820072	0.001727	0.313216		
PDGFRB	K05089	-11.0902	3.511122	8.796661	0.003019	0.376562		
FLT1	K05096	-10.4414	3.479376	8.070282	0.004501	0.406923		
FLT4	K05097	-6.08894	5.005001	6.949418	0.008386	0.466414		
GRIN2C	K05211	-10.3785	2.817897	6.879824	0.008719	0.466956		
CCL3	K05408	-8.19786	4.566051	8.060129	0.004526	0.406923	C-C motif c	LOC110192261
CXCL9	K05416	-10.3095	3.491648	7.962891	0.004776	0.410497		
GDNF	K05452	-9.95643	4.171471	9.358149	0.002221	0.328202		
SLC1A3	K05614	-10.3452	2.830787	6.863069	0.008801	0.466956		

FCER1G	K07983	-7.42538	9.419039	13.69912	0.000215	0.157612		
NCF1	K08011	-5.04173	9.984292	8.352496	0.003853	0.396846		
CCRL2	K08373	-7.96305	7.817002	12.90933	0.000327	0.171082		
P2RY13	K08388	-12.4461	5.732757	11.69771	0.000626	0.218276		
HCAR2	K08402	-6.71016	9.534761	12.05079	0.000518	0.213505		
GPR26	K08411	10.77781	3.196777	10.03228	0.001539	0.340732		
TTBK1	K08815	7.026107	4.707751	10.01452	0.001554	0.340732		
MYO3A	K08834	10.24849	4.251535	13.48367	0.000241	0.158879		
NEK5	K0885	10.64744	3.12126	9.693484	0.00185	0.364531		
ZNF561	K09228	10.01635	2.7831	8.371638	0.003812	0.396846	zinc finger	LOC110193
ZNF251	K09228	10.37125	2.884721	8.870463	0.002899	0.383229	zinc finger	LOC110214
SOX8	K09270	11.22753	3.514473	11.49412	0.000698	0.231646		
SPI1	K09438	-6.73339	8.443892	11.26687	0.000789	0.245861		
IL8	K10030	-5.81208	10.46498	10.41732	0.001249	0.308747		
ZBTB16	K10055	8.273379	3.993338	9.385166	0.002188	0.37908		
CLEC4E	K10059	-5.93611	7.598522	8.926431	0.002812	0.382289		
TBX2	K10176	11.52817	3.968449	13.65123	0.00022	0.157612		
MYH11	K10352	5.33005	5.180803	7.737762	0.005409	0.459665		
MYO15A	K10361	6.002619	5.191853	9.362354	0.002216	0.37908		
ANK2	K10380	7.141117	5.491942	13.36105	0.000257	0.158879		
KIF26B	K10404	7.445518	4.771352	11.10804	0.00086	0.259831		
DNAH5	K10408	6.995315	4.454639	9.120803	0.002528	0.37908		
MAP1B	K10429	6.189529	4.47826	7.655482	0.005661	0.467981		
MCM9	K10738	10.82669	3.306542	10.36466	0.001285	0.312004	DNA helica	LOC110197
MMD2	K11064	11.27823	3.61357	11.86008	0.000574	0.216717		
CUL9	K11970	9.125404	4.479951	13.0726	0.0003	0.168425	cullin-9	LOC110201
ISG15	K12159	-5.50157	9.711829	9.324588	0.002262	0.37908		
GUCY2C	K12320	10.16629	2.694232	8.439283	0.003673	0.393242		
TTN	K12567	8.465235	7.43488	25.72442	3.95E-07	0.001409		
MEFV	K12803	-6.46834	7.641027	9.977322	0.001585	0.342165		
RETN	K13438	-11.3776	6.068172	10.86555	0.00098	0.271327		
Bf-CRAMP	K13916	-7.25115	11.02452	14.36622	0.000151	0.146251	cathelicidir	LOC110216
Bf-CRAMP	K13916	-5.58908	8.926139	9.159973	0.002474	0.37908	cathelicidir	LOC110217
IFIT1	K14217	-7.17787	9.145091	12.78489	0.00035	0.176072	interferon-	LOC110192
SLCO2B1	K14352	6.732859	4.385307	8.355852	0.003845	0.396846		
RPP25	K14525	10.27624	2.796593	8.667593	0.00324	0.385212		
SLC15A5	K14639	9.882867	2.150686	7.636786	0.00572	0.467981		
SHANK2	K15009	7.14377	4.136195	8.164567	0.004273	0.409656		
SIX1	K15614	10.75202	3.505586	10.80758	0.001011	0.271327		
MUC16	K16145	10.58975	3.925609	11.96339	0.000543	0.213505	mucin-16	LOC110203
RGS18	K16449	-12.2421	5.522225	10.78979	0.001021	0.271327		
TIMP1	K16451	-6.04127	6.793091	8.109875	0.004404	0.418032		
PCDHAC1	K16493	10.2039	2.71859	8.505692	0.003541	0.385212	protocadhe	LOC110220
PCDHGC4	K16497	4.725886	7.034222	10.03833	0.001534	0.340732	protocadhe	LOC110220

ABCA12	K05646	-10.705	4.416835	11.22192	0.000809	0.255336		
ABCB1	K05658	-7.96688	3.92795	6.546955	0.010508	0.491544		
PLCB1	K05858	-11.2275	3.508522	8.930664	0.002805	0.365774		
SPTBN5	K06115	-11.1545	5.494331	17.06523	3.62E-05	0.050562		
COL1A1	K06236	-11.6073	4.079409	11.133	0.000848	0.255336		
COL1A2	K06236	-9.97986	3.932976	8.442404	0.003667	0.392234		
COL4A5	K06237	-10.2403	2.895367	6.830432	0.008963	0.466956		
Col6a4	K06238	-8.60087	4.760025	9.927771	0.001629	0.31032	collagen al	LOC110209064
COL6A5	K06238	-11.548	5.216768	15.89054	6.72E-05	0.062065		
HSPG2	K06255	-6.16622	5.367191	8.033592	0.004593	0.406923		
CD36	K06259	-9.61149	3.793938	7.885547	0.004984	0.410738		
SPN	K06477	4.99688	8.711057	7.461855	0.006303	0.43506		
IGSF1	K06512	-10.9645	3.390446	8.398956	0.003755	0.395827	immunoglo	LOC110220196
SIGLEC14	K06549	6.137406	6.321071	7.243157	0.007119	0.442037	sialic acid-b	LOC110219799
IFITM2	K06566	5.392642	7.84318	7.679939	0.005585	0.419336	interferon-	LOC110202976
PLK1	K06631	-10.4736	3.029845	7.248995	0.007096	0.442037		
BTNL2	K06712	-10.3543	2.612397	6.689689	0.009699	0.482388	butyrophili	LOC110194075
BTNL2	K06712	-9.91654	2.987605	6.693712	0.009677	0.482388	butyrophili	LOC110210621
CNTN5	K06763	-11.2782	3.663981	9.3612	0.002217	0.328202		
IGLON5	K06773	-10.6472	2.94746	7.259304	0.007055	0.442037		
ACAN	K06792	-11.3794	4.175631	11.22656	0.000807	0.255336		
VCAN	K06793	4.410334	11.5588	7.145158	0.007518	0.448864		
SLIT1	K06838	-10.8026	3.15152	7.763511	0.005332	0.419336		
SEMA3E	K06840	-10.3112	2.771378	6.784401	0.009197	0.470142		
SRGN	K06849	4.569121	10.0283	7.043432	0.007957	0.454596		
SLIT3	K06850	-11.635	4.146843	11.4408	0.000719	0.255336		
KRT85	K07605	-10.2038	2.759364	6.685665	0.009721	0.482388	keratin, typ	LOC110218182
GJB3	K07622	-11.0696	3.710231	9.260164	0.002343	0.334232		
ADAMTS4	K07764	-11.0287	3.484742	8.673609	0.003229	0.38183		
ARL10	K07958	-11.3258	3.642058	9.359761	0.002219	0.328202		
B3GNT4	K07971	-10.9078	3.197174	7.952067	0.004804	0.410497		
FCER1G	K07983	5.989463	9.419039	10.05558	0.001519	0.31032		
MMP17	K07997	-8.57593	3.856662	6.901357	0.008615	0.466956		
MMP19	K07998	-8.33312	4.097498	7.156074	0.007473	0.448864		
SLC43A3	K08230	-10.3785	2.807087	6.870568	0.008764	0.466956		
CCRL2	K08373	6.588391	7.817002	9.722937	0.00182	0.313216		
P2RY8	K08386	-7.92396	3.932478	6.507541	0.010744	0.498569		
GPR68	K08408	-10.506	3.365964	7.908177	0.004922	0.410738		
GPR84	K08421	-4.56276	6.896508	7.122178	0.007615	0.448864		
ADAMTS17	K08631	-9.53065	3.498336	6.871573	0.008759	0.466956		
SBK1	K08858	-7.71208	4.673417	8.517819	0.003518	0.385571		
PLK3	K08862	4.920907	7.474183	6.621804	0.010076	0.489235		
NPAS1	K09098	-10.4735	3.094713	7.36263	0.006661	0.442037		
ZNF205	K09228	-11.8024	4.116622	11.53098	0.000685	0.255336		

DCHS1	K16507	6.322043	4.739979	8.677113	0.003223	0.385212			ZNF345	K09228	-9.90902	3.508573	7.277293	0.006985	0.442037	zinc finger	LOC110221328
MAP7D3	K16807	9.700666	11.30695	38.73031	4.89E-10	6.65E-06	MAP7 dom	LOC11019	SPI1	K09438	5.745335	8.443892	8.83758	0.002952	0.371622		
SPRY4	K17385	8.491005	4.06703	10.01947	0.00155	0.340732			FKBP6	K09572	-10.5916	3.175275	7.614962	0.00579	0.419336		
HOXC10	K17444	10.57675	3.033447	9.419253	0.002148	0.37908			TMPRSS9	K09640	-11.5341	3.913772	10.44131	0.001233	0.284101		
ACOD1	K17724	-7.63696	10.89394	15.21578	9.60E-05	0.126737			IL8	K10030	4.636594	10.46498	7.340716	0.006742	0.442037		
CRYM	K18258	11.41103	3.666987	12.22329	0.000472	0.213505			CLEC4F	K10060	-10.2761	2.890615	6.856529	0.008833	0.466956	C-type lect	LOC110218459
DYSF	K18261	9.693813	5.572705	19.76606	8.76E-06	0.023832			COLEC12	K10062	-8.16747	5.073547	10.01569	0.001553	0.31032		
NEB	K18267	5.974449	5.169835	9.229383	0.002382	0.37908			LGALS9C	K10093	-9.43372	3.649997	7.371513	0.006628	0.442037	galectin-9C	LOC110192174
TDRD15	K18405	7.074194	4.285679	8.581732	0.003396	0.385212			TLR9	K10161	-10.2761	2.918268	6.889993	0.00867	0.466956		
TDRD5	K18407	8.099112	3.425609	7.461279	0.006305	0.48991			MYH11	K10352	-11.5143	5.180803	15.85434	6.85E-05	0.062065		
DUSP9	K18498	10.16632	2.677811	8.422761	0.003707	0.393462			MYH7B	K10352	-12.4918	4.795777	15.63153	7.70E-05	0.064225		
TCHH	K18626	5.315557	7.240832	12.55207	0.000396	0.192295			MYO7A	K10359	-6.851	5.166146	8.720794	0.003147	0.378659		
SLF1	K18757	7.037173	4.284279	8.519582	0.003514	0.385212			MYO15A	K10361	-9.223	5.191853	12.65762	0.000374	0.169623		
SUN2	K19347	12.37752	4.630274	18.93194	1.36E-05	0.026709	SUN domai	LOC11020	FMN1	K10367	-9.74657	3.566068	7.229595	0.007173	0.442037	formin-1	LOC110214857
JPH2	K19530	10.87302	3.852662	12.12511	0.000498	0.213505			ANK2	K10380	-7.05316	5.491942	10.13574	0.001455	0.309069		
MACF1	K19827	9.329378	3.395018	8.824245	0.002973	0.383229	microtubul	LOC11021	DST	K10382	-9.72186	3.224148	6.89865	0.008628	0.466956	dystonin-lil	LOC110200818
SYT2	K19902	6.957191	4.246629	8.248969	0.004079	0.407767			DST	K10382	-5.96444	5.065925	6.9377	0.008441	0.466414	dystonin	LOC110214423
TNFAIP2	K19989	-6.22513	11.44258	11.94963	0.000547	0.213505			KIF24	K10393	-8.88901	4.142827	8.087044	0.004459	0.406923		
TAGAP	K20654	-4.87421	9.034811	7.585355	0.005886	0.469552			KIF2C	K10393	-10.3783	2.931444	6.998789	0.008158	0.462188		
AEBP1	K21392	7.890164	4.069787	9.028078	0.002659	0.379163			KIFC1	K10405	-10.9858	3.653626	9.02384	0.002666	0.355337		
CYBB	K21421	-6.06812	6.660558	7.781063	0.005281	0.453255	cytochrom	LOC11020	DNAH1	K10408	-6.6192	4.764218	7.238908	0.007135	0.442037		
MYRIP	K22237	11.19665	3.449487	11.25229	0.000796	0.245861			DNAH17	K10408	-7.70304	4.9047	9.196265	0.002426	0.336575		
Pseudo	N/A	-5.99608	8.098983	9.527548	0.002025	0.37908	Pseudo	LOC11021	MAP2	K10430	-8.09668	4.517613	8.586251	0.003388	0.385571		
uncharacte	N/A	-5.73974	8.072795	8.866582	0.002905	0.383229	uncharacte	LOC11021	KLHL5	K10442	-10.7935	3.416784	8.288682	0.00399	0.4019		
IGKC	N/A	-5.45843	8.376952	8.548245	0.00346	0.385212	Ig kappa ch	LOC11020	ZBTB47	K10518	-10.9406	4.179013	10.66603	0.001092	0.269878		
IGHA2	N/A	-5.16436	8.93868	8.180992	0.004234	0.409656	immunoglo	LOC11019	TRIM63	K10655	-10.7523	3.367866	8.143273	0.004323	0.406923		
Pseudo	N/A	5.976445	4.829452	8.069042	0.004504	0.419449	Pseudo	LOC11019	GINS4	K10735	-10.9496	3.593452	8.844418	0.002941	0.371622		
Pseudo	N/A	6.355792	6.484914	14.56933	0.000135	0.141404	Pseudo	LOC11022	DNA2	K10742	-10.7011	3.101779	7.57927	0.005906	0.419336		
Pseudo	N/A	6.498159	8.183468	18.90574	1.38E-05	0.026709	Pseudo	LOC11020	RRM2	K10808	-9.54925	3.27525	6.836729	0.008932	0.466956		
uncharacte	N/A	6.947519	4.075819	7.54073	0.006033	0.476945	uncharacte	LOC11021	ASXL3	K11471	-11.4287	3.743073	9.753664	0.00179	0.313216		
uncharacte	N/A	7.344245	4.175125	8.617278	0.003331	0.385212	uncharacte	LOC11021	CENPE	K11498	-9.09729	4.804953	10.74913	0.001044	0.268071		
Pseudo	N/A	7.762366	3.985594	8.605374	0.003353	0.385212	Pseudo	LOC11020	CENPF	K11499	-9.03182	5.20905	10.81542	0.001007	0.268071		
uncharacte	N/A	7.831026	8.700448	25.62924	4.15E-07	0.001409	uncharacte	LOC11020	SGO2	K11581	-10.4738	2.951746	7.114699	0.007647	0.448864		
Pseudo	N/A	8.163014	4.228648	10.16582	0.001431	0.335524	Pseudo	LOC11019	ISG15	K12159	5.735535	9.711829	9.677633	0.001866	0.313216		
Pseudo	N/A	8.553402	3.375712	7.891769	0.004967	0.438187	Pseudo	LOC11021	HECW1	K12167	-12.0912	4.557909	13.95468	0.000187	0.120867		
uncharacte	N/A	9.008606	9.003446	31.54293	1.96E-08	0.000133	uncharacte	LOC11020	GUCY1A2	K12318	-11.7657	4.737209	14.17347	0.000167	0.119383		
Pseudo	N/A	9.1945	3.260902	8.316794	0.003929	0.401678	Pseudo	LOC11021	MRVI1	K12337	-11.6352	4.013074	10.91873	0.000952	0.268071		
uncharacte	N/A	9.55104	5.12833	17.32221	3.16E-05	0.053674	uncharacte	LOC11021	NEU4	K12357	-10.7079	3.124378	7.626992	0.005751	0.419336		
uncharacte	N/A	9.723698	2.578008	7.842571	0.005104	0.444883	uncharacte	LOC11020	MYBPC2	K12558	-9.89037	3.662679	7.587741	0.005878	0.419336		
Pseudo	N/A	9.807812	2.223046	7.628339	0.005747	0.467981	Pseudo	LOC11020	TTN	K12567	-7.51108	7.43488	16.87738	3.99E-05	0.050562		
uncharacte	N/A	9.915801	3.912245	11.32691	0.000764	0.245861	uncharacte	LOC11021	MYBPC3	K12568	-11.1485	4.64868	12.83508	0.00034	0.169623		
uncharacte	N/A	9.95368	2.224928	7.774453	0.0053	0.453255	uncharacte	LOC11021	ISG20	K12579	4.620998	9.11343	6.851008	0.008861	0.466956		
uncharacte	N/A	10.04735	2.544398	8.16224	0.004278	0.409656	uncharacte	LOC11020	CCL4	K12964	-10.175	2.604589	6.538842	0.010556	0.49155	C-C motif c	LOC110221243
Pseudo	N/A	10.41073	2.995023	9.128699	0.002517	0.37908	Pseudo	LOC11020	CELF6	K13207	-7.05622	4.813629	7.889696	0.004973	0.410738		

uncharacte	N/A	10.47393	2.886102	8.988631	0.002717	0.379163	uncharacte	LOC11020	ABHD1	K13696	-10.5633	3.020914	7.311952	0.006851	0.442037		
uncharacte	N/A	10.60517	2.884832	9.142639	0.002498	0.37908	uncharacte	LOC11021	PIP5KL1	K13712	-11.0477	4.288221	11.24469	0.000799	0.255336		
PLAC8	No KO	-8.16638	7.04413	11.74385	0.000611	0.218276			BEST1	K13878	-10.0663	3.554951	7.844669	0.005098	0.415097		
JCHAIN	No KO	-6.33214	7.069047	9.165493	0.002467	0.37908			MARCO	K13884	-9.61217	3.671977	7.602201	0.005831	0.419336		
NGP	No KO	-5.5637	7.012403	7.61691	0.005784	0.468096	neutrophili	LOC11021	KLHL33	K13957	-10.8743	3.241012	8.00135	0.004675	0.406923		
VSIR	No KO	-5.54128	7.803693	8.287248	0.003993	0.405219			MOV10L1	K13983	-11.0496	3.430699	8.572225	0.003414	0.385571		
RIN3	No KO	-5.48938	7.723385	8.180555	0.004235	0.409656			IFIT1	K14217	6.434765	9.145091	10.82403	0.001002	0.268071	interferon-	LOC110192724
TMEM140	No KO	-5.47182	7.270937	7.665994	0.005628	0.467981			SCRN1	K14358	-11.9041	4.196736	12.007	0.00053	0.212066		
IFIT3	No KO	-5.14017	10.59581	8.826321	0.00297	0.383229	interferon-	LOC11019	SLC5A10	K14390	-10.3986	2.706923	6.803952	0.009097	0.467943		
TENM4	No KO	5.420449	5.556261	8.983661	0.002725	0.379163			SULF1	K14607	-10.62	3.026472	7.371534	0.006628	0.442037		
CFAP46	No KO	5.50601	5.085559	7.881622	0.004995	0.438187			CUBN	K14616	-11.313	3.68295	9.44635	0.002116	0.327986		
FAM71A	No KO	5.558894	7.594589	14.17268	0.000167	0.151282	protein FAI	LOC11020	SLC26A10	K14707	-8.58098	4.157284	7.528525	0.006074	0.423549		
WDR87	No KO	6.02977	5.886801	11.63799	0.000646	0.219761	WD repeat	LOC11021	SLC9A2	K14722	-10.2032	3.061236	7.064413	0.007865	0.451199	sodium/hy	LOC110193056
ERICH3	No KO	6.145232	4.924893	8.833914	0.002958	0.383229			SHANK1	K15009	-8.07582	4.25931	7.638572	0.005715	0.419336		
ZNF608	No KO	6.159619	4.509178	7.661862	0.005641	0.467981			PIF1	K15255	-10.1662	2.761133	6.656028	0.009884	0.486922		
ZNF536	No KO	6.295053	4.7862	8.6215	0.003323	0.385212			MUC16	K16145	-9.46589	5.739134	15.55283	8.03E-05	0.064225	mucin-16-l	LOC110223371
NYAP1	No KO	6.316053	4.37737	7.579395	0.005905	0.469552			TIMP1	K16451	5.603382	6.793091	7.120887	0.007621	0.448864		
FAM71B	No KO	6.380576	6.228985	13.85221	0.000198	0.157612	protein FAI	LOC11019	PCNT	K16481	-10.6052	2.884832	7.13237	0.007572	0.448864	RNA-perice	LOC110215026
LRRIQ1	No KO	6.742808	4.141553	7.525461	0.006085	0.478223			PCNT	K16481	-10.5634	2.955615	7.198239	0.007299	0.446821	RNA-perice	LOC110203849
GRID2IP	No KO	6.789374	4.622525	9.258852	0.002344	0.37908			STARD9	K16491	-6.71301	5.720607	10.17083	0.001427	0.308056		
FBN3	No KO	7.626735	4.07851	8.662845	0.003249	0.385212			PCDHGA2	K16495	-4.34717	7.034222	6.963817	0.008319	0.466414	protocadh	LOC110220871
NAV3	No KO	7.739044	4.154658	9.12797	0.002518	0.37908			PCDHGA5	K16495	-10.3774	3.339566	7.722799	0.005454	0.419336	protocadh	LOC110220822
DNHD1	No KO	7.90526	4.48602	10.94022	0.000941	0.271327			CDHR1	K16501	-11.0073	3.625938	8.98048	0.00273	0.360335		
RIMBP3	No KO	8.402338	4.53588	11.94037	0.00055	0.213505	RIMS-bindi	LOC11021	CDHR5	K16505	-10.8148	4.331034	11.09964	0.000864	0.255336		
MPP2	No KO	8.57907	3.390567	7.953828	0.0048	0.437504			PTPRG	K16667	-6.71437	4.564771	6.880804	0.008714	0.466956		
PLEKHH1	No KO	8.695365	3.605264	8.631529	0.003305	0.385212			TMIGD2	K16668	-10.041	3.177634	7.116076	0.007641	0.448864		
GULP1	No KO	8.945499	4.81894	13.95326	0.000188	0.157612			MAP7D3	K16807	-14.3824	11.30695	46.92664	7.42E-12	8.4E-08	MAP7 dom	LOC110196501
ZNF260	No KO	9.075333	3.231609	8.100176	0.004427	0.418032	zinc finger	LOC11019	ANKRD7	K17299	-6.92518	4.964037	8.25072	0.004075	0.405415	ankyrin rep	LOC110203999
FAM170B	No KO	9.190228	5.051315	15.09101	0.000103	0.126737	protein FAI	LOC11020	SPRY4	K17385	-11.8132	4.06703	11.34409	0.000757	0.255336		
TOGARAM	No KO	9.413404	3.416258	8.990855	0.002714	0.379163			SYTL3	K17598	-10.2396	3.772889	8.528402	0.003497	0.385571		
FBN2	No KO	9.500202	2.456688	7.495375	0.006187	0.483483	fibrillin-2	LOC11019	RGL1	K17635	-10.3775	3.284691	7.616784	0.005784	0.419336		
ZNF345	No KO	9.530034	3.508573	9.394782	0.002177	0.37908	zinc finger	LOC11022	ACOD1	K17724	5.393933	10.89394	9.281219	0.002316	0.334232		
VSTM5	No KO	9.787616	2.601655	7.934047	0.004852	0.437504			TNS4	K18080	-10.7009	3.214457	7.788812	0.005258	0.419336		
CCDC87	No KO	9.823856	2.543507	7.919803	0.004891	0.437504			CRYM	K18258	-11.411	3.666987	9.512752	0.002041	0.327986		
BPIFB4	No KO	9.917627	2.442565	7.928192	0.004868	0.437504	BPI fold-co	LOC11021	NEB	K18267	-9.05175	5.169835	12.3032	0.000452	0.186413		
CFAP54	No KO	10.00896	3.989681	11.80102	0.000592	0.217654			TDRD6	K18405	-12.5602	4.870736	16.10661	5.99E-05	0.062065		
MRO	No KO	10.12587	3.410959	9.713684	0.00183	0.364531			PDE8B	K18437	-11.8518	4.301344	12.41021	0.000427	0.181531		
CFAP206	No KO	10.31124	2.727594	8.634182	0.0033	0.385212			ANLN	K18621	-7.83485	4.336163	7.583445	0.005892	0.419336		
CAMK2N2	No KO	10.32833	2.697872	8.634658	0.003299	0.385212			SUN2	K19347	-7.08987	5.271254	8.917973	0.002825	0.365774	SUN domai	LOC110199948
LRRC7	No KO	10.34391	3.384159	9.936499	0.001621	0.34437			ART5	K19977	-11.0274	4.070246	10.37264	0.001279	0.289949		
MATN1	No KO	10.44252	3.018781	9.21761	0.002398	0.37908			LRP1B	K20049	-10.3058	4.677622	11.85013	0.000577	0.224102		
LDLRAD2	No KO	10.50436	2.921537	9.07921	0.002586	0.37908			LRP4	K20051	-6.16254	5.142962	7.433858	0.006402	0.439648		
CD164L2	No KO	10.59915	3.11102	9.611303	0.001934	0.375761			DOK2	K20234	-7.76728	4.170703	6.933181	0.008463	0.466414		
KY	No KO	10.75286	3.070436	9.703074	0.00184	0.364531			ARHGAP21	K20315	-5.4399	5.667789	7.370105	0.006633	0.442037	RNA-rho G	LOC110204120

UNC5CL	No KO	10.80247	3.242965	10.17544	0.001424	0.335524		
WFIKKN1	No KO	10.83212	3.08214	9.826686	0.001721	0.354467		
ZNF474	No KO	11.58483	4.357143	15.78719	7.09E-05	0.107177	zinc finger	LOC11022

ARHGAP21	K20315	-10.5633	3.017187	7.305409	0.006876	0.442037	rho GTPase	LOC110220892
SEC16B	K20353	-10.4106	3.063634	7.252114	0.007083	0.442037		
ARHGAP28	K20639	-10.8912	3.227941	7.992382	0.004699	0.406923		
ECT2	K20704	-10.3782	2.996632	7.108542	0.007673	0.448864		
PANX2	K20857	-10.3262	4.546403	11.17547	0.000829	0.255336		
NLRP1	K20865	-8.45812	4.911816	9.911334	0.001643	0.31032		
ANTXRL	K20909	-11.0496	3.393647	8.4895	0.003573	0.385571		
NOTCH4	K20996	-6.89679	4.766919	7.660742	0.005645	0.419336		
MUC5AC	K21125	-6.45417	4.740078	6.933157	0.008463	0.466414		
CYBB	K21421	5.513959	6.660558	6.611097	0.010136	0.489235	cytochrom	LOC110203146
USH1G	K21878	-10.8741	3.356134	8.236469	0.004107	0.405415		
FAM20C	K21958	-10.5622	3.492556	8.219863	0.004144	0.405415		
UNC45B	K21991	-10.896	3.937208	9.765941	0.001778	0.313216		
MYRIP	K22237	-11.1966	3.449487	8.762574	0.003076	0.378659		
CLSTN3	K22661	-10.8742	3.290547	8.098568	0.004431	0.406923		
KANK3	K22808	-10.3453	2.780146	6.820011	0.009016	0.467327		
GPR179	K22961	-12.0194	4.347647	12.86554	0.000335	0.169623		
UCN	K23142	-10.3557	2.620757	6.697845	0.009655	0.482388		
PMFBP1	K23223	-7.04819	4.356282	6.700359	0.009641	0.482388		
CUNH16or	N/A	-10.8132	3.106845	7.693638	0.005543	0.419336		
CUNH16or	N/A	-11.5356	3.930852	10.50239	0.001193	0.282938		
CUNH1orf1	N/A	-9.3884	4.701689	10.61902	0.00112	0.271882		
CUNH8orf4	N/A	-10.5135	2.924996	7.104216	0.007692	0.448864		
Pseudo	N/A	-14.2042	6.484914	28.63376	8.77E-08	0.000298	Pseudo	LOC110220304
Pseudo	N/A	-11.288	3.691364	9.440864	0.002123	0.327986	Pseudo	LOC110220742
Pseudo	N/A	-11.278	3.745321	9.59484	0.001952	0.32366	Pseudo	LOC110195951
Pseudo	N/A	-11.2229	4.844789	13.7742	0.000206	0.12195	Pseudo	LOC110215492
Pseudo	N/A	-10.9854	3.819205	9.497281	0.002058	0.327986	Pseudo	LOC110213973
Pseudo	N/A	-10.8023	3.320226	8.091237	0.004449	0.406923	Pseudo	LOC110199229
Pseudo	N/A	-10.7064	3.834606	9.227502	0.002385	0.334293	Pseudo	LOC110199404
Pseudo	N/A	-10.6745	3.134749	7.615834	0.005787	0.419336	Pseudo	LOC110197170
Pseudo	N/A	-10.5333	3.368135	7.93101	0.004861	0.410497	Pseudo	LOC110223090
Pseudo	N/A	-10.0469	2.790403	6.581158	0.010308	0.49007	Pseudo	LOC110223369
Pseudo	N/A	-9.87907	4.189003	9.151818	0.002485	0.341365	Pseudo	LOC110203424
Pseudo	N/A	-9.86663	3.953852	8.595784	0.00337	0.385571	Pseudo	LOC110194165
Pseudo	N/A	-9.4871	4.276046	9.246812	0.00236	0.334232	Pseudo	LOC110218211
Pseudo	N/A	-8.72185	4.398933	7.704993	0.005508	0.419336	Pseudo	LOC110210617
Pseudo	N/A	-8.20258	4.630274	9.096691	0.002562	0.34484	Pseudo	LOC110203563
Pseudo	N/A	-6.66947	8.183468	15.3299	9.03E-05	0.068249	Pseudo	LOC110204714
Pseudo	N/A	-5.95065	5.65995	8.299593	0.003966	0.4019	Pseudo	LOC110223355
Pseudo	N/A	5.17847	8.098983	7.555806	0.005983	0.419336	Pseudo	LOC110211833
uncharacte	N/A	-16.7526	9.003446	45.9266	1.24E-11	8.4E-08	uncharacte	LOC110204397
uncharacte	N/A	-16.4426	8.700448	44.13776	3.08E-11	1.4E-07	uncharacte	LOC110204395
uncharacte	N/A	-12.723	5.138558	17.72914	2.55E-05	0.043335	uncharacte	LOC110203791

uncharacte	N/A	-11.5393	3.979246	10.67878	0.001084	0.269878	uncharacte	LOC110220800
uncharacte	N/A	-11.4755	3.791218	9.960619	0.0016	0.31032	uncharacte	LOC110195385
uncharacte	N/A	-11.3071	3.613685	9.268179	0.002332	0.334232	uncharacte	LOC110222943
uncharacte	N/A	-11.0296	3.422315	8.536557	0.003482	0.385571	uncharacte	LOC110207231
uncharacte	N/A	-10.9294	3.452057	8.501652	0.003549	0.385571	uncharacte	LOC110221959
uncharacte	N/A	-10.7934	3.4315	8.322209	0.003917	0.400483	uncharacte	LOC110202211
uncharacte	N/A	-10.6742	3.283771	7.895776	0.004956	0.410738	uncharacte	LOC110211369
uncharacte	N/A	-10.6291	2.936832	7.22657	0.007185	0.442037	uncharacte	LOC110211753
uncharacte	N/A	-10.3112	2.727299	6.747683	0.009389	0.476333	uncharacte	LOC110201453
uncharacte	N/A	-10.1664	2.622687	6.544966	0.01052	0.491544	uncharacte	LOC110202919
uncharacte	N/A	-10.1663	2.685533	6.594896	0.010229	0.489235	uncharacte	LOC110220146
uncharacte	N/A	-8.90348	3.696645	6.895799	0.008641	0.466956	uncharacte	LOC110203721
uncharacte	N/A	-7.72141	4.357199	6.761078	0.009318	0.474542	uncharacte	LOC110192933
uncharacte	N/A	-7.48149	4.666827	7.999286	0.004681	0.406923	uncharacte	LOC110218577
uncharacte	N/A	-7.12177	4.576328	7.255015	0.007072	0.442037	uncharacte	LOC110207158
uncharacte	N/A	-7.07858	4.547116	6.835509	0.008938	0.466956	uncharacte	LOC110219148
uncharacte	N/A	-6.82099	5.12833	8.57596	0.003407	0.385571	uncharacte	LOC110217275
uncharacte	N/A	-6.22039	5.575924	8.739342	0.003115	0.378659	uncharacte	LOC110204294
uncharacte	N/A	5.510721	8.072795	8.17877	0.004239	0.406923	uncharacte	LOC110213904
uncharacte	N/A	6.671029	5.94936	7.294007	0.00692	0.442037	uncharacte	LOC110214822
ADAMTSL1	no KO	-7.91445	4.170084	7.191079	0.007328	0.446821		
ALS2CR12	no KO	-11.3964	3.823152	9.975735	0.001587	0.31032		
Bf-CRAMP	no KO	6.731994	11.02452	12.80191	0.000346	0.169623	cathelicidir	LOC110216987
BHMG1	no KO	-8.61239	4.384852	8.679784	0.003219	0.38183		
CCDC136	no KO	-8.96344	4.12811	8.134956	0.004343	0.406923		
CCDC96	no KO	-10.3631	2.751643	6.815377	0.009039	0.467327		
CCIN	no KO	-10.3109	2.9252	6.931053	0.008473	0.466414		
CFAP46	no KO	-7.40658	5.085559	9.405964	0.002164	0.328202		
CFAP54	no KO	-11.7399	3.989681	10.96027	0.000931	0.268071		
CLRN3	no KO	-10.1663	2.684418	6.594003	0.010234	0.489235		
CMTM7	no KO	-10.3449	2.991085	7.070247	0.007839	0.451199		
CMYA5	no KO	-5.89817	5.590931	8.121263	0.004376	0.406923		
CPAMD8	no KO	-7.52061	4.693654	8.351595	0.003855	0.399392		
DCDC1	no KO	-10.4731	3.294787	7.725988	0.005444	0.419336	doublecort	LOC110192597
DNHD1	no KO	-12.2268	4.48602	13.87451	0.000196	0.120867		
FAM71A	no KO	-8.66611	7.594589	20.80486	5.09E-06	0.011541	protein FAI	LOC110204398
FAM71B	no KO	-10.6184	6.228985	20.12955	7.25E-06	0.014076	protein FAI	LOC110193420
FRMPD2	no KO	-8.36755	4.197782	7.710604	0.005491	0.419336		
FRMPD4	no KO	-12.5219	4.833198	15.85931	6.83E-05	0.062065		
GPM6B	no KO	-8.00933	4.655692	8.429686	0.003692	0.392234		
GRIA1	no KO	-6.46868	4.850723	6.663412	0.009843	0.486672		
HAVCR2	no KO	5.972461	6.930189	7.937212	0.004844	0.410497		
IFIT3	no KO	4.705312	10.59581	7.562566	0.005961	0.419336	interferon-	LOC110198959
ITIH4	no KO	-9.45649	3.782194	7.414418	0.006472	0.442037	inter-alpha	LOC110197543

KIAA1324L	no KO	-9.48406	3.392233	6.620829	0.010081	0.489235		
KIAA1755	no KO	-11.3297	3.915538	10.20646	0.0014	0.307035		
LOXHD1	no KO	-11.2781	3.697293	9.447058	0.002116	0.327986	lipoxygena	LOC110221375
LTF	no KO	5.117372	8.836033	7.860398	0.005054	0.413981	lactotransf	LOC110208182
MEGF11	no KO	-10.6813	4.450572	11.34412	0.000757	0.255336		
PATL2	no KO	-5.95388	5.301088	7.241959	0.007123	0.442037		
PLAC8	no KO	7.620532	7.04413	10.48047	0.001207	0.282938		
PLEKHA4	no KO	-11.4599	3.875321	10.22231	0.001388	0.307035		
PLEKHO1	no KO	-10.3108	2.982331	7.026015	0.008035	0.457118		
PRR36	no KO	-8.69798	3.879898	6.799496	0.00912	0.467943	proline-rich	LOC110196988
PRR5L	no KO	-11.7911	4.396486	12.77812	0.000351	0.169623		
PRX	no KO	-9.24645	3.688987	7.064475	0.007864	0.451199		
PTCHD3	no KO	-10.239	3.388834	7.686118	0.005566	0.419336		
RCOR2	no KO	-10.5342	2.938835	7.144318	0.007522	0.448864		
RETN	no KO	12.5698	6.068172	12.66811	0.000372	0.169623		
SAMSN1	no KO	-4.90184	5.975463	6.548104	0.010501	0.491544		
SCHIP1	no KO	-10.4736	3.040705	7.267884	0.007021	0.442037	schwannor	LOC110210081
SLITRK5	no KO	-10.7012	3.060568	7.504239	0.006157	0.427108		
SMOC2	no KO	-10.599	3.180858	7.632065	0.005735	0.419336		
SYNDIG1	no KO	-10.592	2.985742	7.275317	0.006992	0.442037		
TCF23	no KO	-10.5331	3.439009	8.077474	0.004483	0.406923		
TENM4	no KO	-13.2446	5.556261	21.10683	4.35E-06	0.011541		
TEX15	no KO	-11.5791	3.984833	10.747	0.001045	0.268071		
TLCD2	no KO	-10.4928	4.195124	9.982423	0.001581	0.31032		
TRIL	no KO	-10.5319	3.860822	9.105959	0.002549	0.34484		
TTC25	no KO	-7.07575	4.875089	8.232225	0.004116	0.405415		
UNC80	no KO	-6.86453	4.385237	6.592178	0.010245	0.489235		
WDR87	no KO	-9.75705	5.886801	16.83076	4.09E-05	0.050562	WD repeat	LOC110219911
WDR93	no KO	-10.047	2.7732	6.567298	0.010389	0.490976		
WFIKKN1	no KO	-10.8321	3.08214	7.660905	0.005644	0.419336		
XIRP2	no KO	-11.7023	4.134975	11.47839	0.000704	0.255336		
ZCCHC24	no KO	-10.1664	2.648606	6.565464	0.010399	0.490976		
ZNF469	no KO	-7.83261	5.753497	12.55208	0.000396	0.173685		
